# Progress in Photo-Therapy

**Published:** 1903-06-06

**Authors:** Sydney Stephenson


### Editor's Letter-Box.

**[Our Correspondents are reminded that prolixity is a great bar to publication, and that brevity of style and conciseness of statement greatly facilitate early insertion.]**

### PROGRESS IN PHOTO-THERAPY.

Mr. Sydney Stephenson writes: "Sir,—The section on progress in photo-therapy in your issue of May 23rd contains an erroneous statement, which I beg you will allow me to correct. The report says: 'Stephenson and Walsh first reported the possibility of curing trachoma or "granular lids" with the *x*-rays or high-frequency currents, and Mayou substantiates this as far as the *x*-rays are concerned, though the methods differ somewhat.' It cannot be too clearly understood that the first case of trachoma treated by the *x*-rays was shown at the Ophthalmological Society by Mr. Mayou on June 12th, 1902, and published in the twenty-second volume of the *Transactions* of that Society. This antedated the communication of Dr. Walsh and myself by several months. On the other hand, Dr. Walsh and I claim priority in being the first to apply the high-frequency current to cure trachoma."

[A correction of the statement to which Mr. Stephenson refers appeared in our last week's issue.—Ed. The Hospital.]

